# Efficacy of Clarithromycin Depends on the Bacterial Density in Clarithromycin-Heteroresistant *Helicobacter pylori* Infections: An *In Situ* Detected Susceptibility and Quantitative Morphometry-Based Retrospective Study

**DOI:** 10.3389/pore.2021.1609863

**Published:** 2021-06-29

**Authors:** Jewel Ju Ea Kim, Ildikó Kocsmár, György Miklós Buzás, Ildikó Szirtes, Orsolya Rusz, Csaba Diczházi, Attila Szijártó, István Hritz, Zsuzsa Schaff, András Kiss, Éva Kocsmár, Gábor Lotz

**Affiliations:** ^1^ 2nd Department of Pathology, Semmelweis University, Budapest, Hungary; ^2^ Department of Gastroenterology, Ferencváros Health Centre, Budapest, Hungary; ^3^ Department of Pharmacy, Péterfy Hospital - National Institute of Traumatology, Budapest, Hungary; ^4^ Department of Pathology, Péterfy Hospital - National Institute of Traumatology, Budapest, Hungary; ^5^ 1st Department of Surgery and Interventional Gastroenterology, Semmelweis University, Budapest, Hungary

**Keywords:** fluorescence *in situ* hybridization, *Helicobacter pylori*, clarithromycin, antibiotic resistance, heteroresistance, bacterial density

## Abstract

The global rise in clarithromycin (Cla) resistance is considered to be the main contributor of *Helicobacter pylori* (*Hp*) eradication failures. In nearly half of the Cla-resistant *Hp* infections, Cla-susceptible bacteria are simultaneously present with the Cla-resistant ones (Cla-heteroresistance). The proportion of resistant bacteria in the bacterial population (R-fraction) and its predictive role for the use of Cla-based therapies in Cla-heteroresistant infections has not yet been investigated. Our retrospective study analyzed gastric biopsy samples of 62 *Hp*-positive patients with Cla-heteroresistant infection. Fluorescence *In Situ* Hybridization technique was used to visualize the coexistence of resistant and susceptible bacteria within one tissue sample. R-fraction was quantified on multichannel microimages by digital morphometry. Resistant bacteria had a patchy distribution within the whole bacterial population causing high diversity among the investigated areas. Patients were subdivided into two major groups according to whether a Cla-based eradication attempt was conducted before or after the biopsy sampling. R-fraction was significantly lower among cases having only one previous Cla-based eradication attempt vs. those that had multiple previous eradications, including at least one Cla-containing therapy (0.41 vs. 0.89, *p* = 0.0308). Majority of the patients without previous eradication attempt had successful eradication with Cla-containing regimen (59.26%), verified by a negative ^13^C-urea breath test or control biopsy. Multivariable model indicated that the therapeutic outcome using Cla-based regimens depended on the bacterial density rather than the R-fraction. Our study raises the potential use of Cla-containing eradication therapies in certain Cla-heteroresistant *Hp* infections, taking into account the possible predictive role of bacterial density.

## Introduction

The prevalence of infection with *Helicobacter pylori* (*H. pylori*), a gram negative, spiral shaped bacterium has reached ∼50% of the population worldwide (1). Clarithromycin is one of the most potent antibiotic used in the eradication of *H. pylori*. It is an acid-stable antimicrobial drug, and along with its active metabolite, 14-hydroxy-clarithromycin, it concentrates in gastric juice and mucosa. The most frequently documented side effects of clarithromycin are usually mild, including nausea, diarrhea, and taste disturbance (2,3). Several studies proved its synergistic effect when used in combination and confirmed the usefulness of clarithromycin-based triple therapy in eradicating *H. pylori* (4–6). This triple therapy containing a combination of two antimicrobial agents (clarithromycin combined with amoxicillin or metronidazole) and a proton pump inhibitor has been considered the most effective first line therapy for *H. pylori* infections (7). However, due to the rise in resistance among the commonly used antibiotics for *H. pylori* infections, particularly clarithromycin (Cla), successful eradication has been a challenge in numerous cases (8–10). Therefore, the latest international guidelines have strongly recommended the need for routine antibiotic susceptibility testing (11,12). Multiple methods are used in detecting resistant bacteria, notably polymerase chain reaction (PCR), which is the most widely used method in detecting bacterial point mutations resulting in Cla-resistance (13,14). The European Committee for Antimicrobial Susceptibility Testing (EUCAST) recommends the use of E-test and minimum inhibitory concentration (MIC) breakpoints for testing *H. pylori* susceptibility (15).

These widely used *H. pylori* Cla-resistance detection methods are however not adapted to reveal intraniche heteroresistance, represented by the coexistence of susceptible and resistant microbes in the same gastric mucosal site (16,17), as they only give a yes or no result in routine settings. Another manifestation of heteroresistance is known as interniche heteroresistance, where isolates with different resistance attributes (susceptible, resistant, or mixed) are found within different sites of the stomach, resulting in an underestimation of the actual antimicrobial resistance, as it is difficult to detect resistance when the isolates are not uniformly distributed in the gastric mucosa (16–18). Fluorescence *in situ* hybridization (FISH) method is capable of revealing individual *H. pylori* bacteria carrying Cla-resistance causing point mutations (19), as well as visualizing the coexistence of susceptible and resistant bacteria within one biopsy sample (18,20).

Our research group has recently published an improved concept on *H. pylori* heteroresistance, proposing that Cla-heteroresistant cases form a distinct subgroup of the *H. pylori* infections with intermediate characteristics compared to the susceptible and homoresistant cases. Accordingly, unlike homoresistant infections, heteroresistant infections are not closely related to prior unsuccessful eradication attempts, and *H. pylori* eradication with clarithromycin-containing regimens might be successful in more than half of the Cla-heteroresistant infections (18).

As there is no standard protocol for diagnosing intraniche heteroresistance nor are there standard treatment guidelines for *H. pylori* heteroresistant infections (21), the aim of our retrospective study was to investigate the Cla-heteroresistant *H. pylori* infections in more detail, with a particular interest in the efficacy of Cla-based eradication therapies. Our hypothesis was that eradication success with Cla-containing regimens depends on the proportion of susceptible and resistant bacteria in the *H. pylori* population of the stomach. Therefore, our primary aim was to measure the proportion of resistant bacteria (R-fraction) within each sample, and correlate it to the efficacy of *H. pylori* eradication.

## Methods

### Patient Selection, Clinical Data, and Ethics

Patients who underwent upper endoscopy procedure with mapping biopsy taken from the antral (*n* = 3) and oxyntic (*n* = 2) compartment of gastric mucosa at the 1st Department of Surgery and Interventional Gastroenterology, Semmelweis University, or at the Péterfy Hospital, Budapest, were selected for this retrospective cross-sectional study from the period of 2005–2019. The inclusion criteria were Cla-heteroresistant *H. pylori* infection confirmed by FISH method, and sufficient anamnestic and treatment outcome data in the institutional electronic database. Histopathological workup was performed at the 2nd Department of Pathology, Semmelweis University, Budapest. Clinical data of the patients were collected from the institutional electronic patient register. The outcome of Cla-based *H-pylori* eradication was evaluated six weeks post-therapy by ^13^C-urea breath test (UBT) or control biopsy.

**FIGURE 1:**
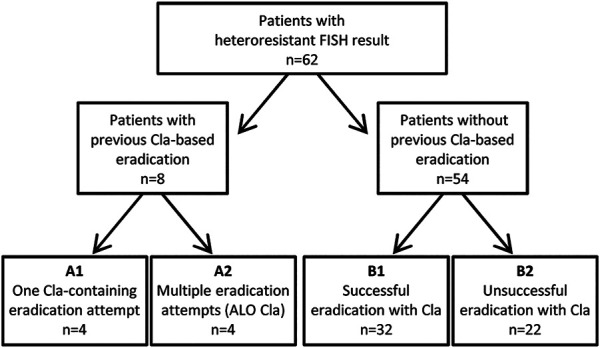
Case selection and subgroups of patients in the study. FISH, Fluorescence *In Situ* Hybridization; Cla, clarithromycin; ALO Cla, At Least One eradication attempt with Clarithromycin-based regimen.

A total of 62 patients met these criteria, including 8 patients with previous eradication history and 54 patients who had never undergone eradication previously. These two basic patient groups were divided into subgroups according to their anamnestic or follow-up data in the electronic register. Among the patients with previous eradication history, four patients had one Cla-based eradication attempt (A1 subgroup) while the other four patients had history of multiple previous eradication treatments including at least one Cla-based eradication attempt (A2 subgroup). Cases without previous eradication attempts were subdivided according to the success of the consequent Cla-containing therapy as follows: B1 subgroup is composed of cases with negative UBT-result or control biopsy (*n* = 32) while B2 subgroup comprised of patients having positive UBT-result or control biopsy (*n* = 22). The subgroups are depicted in Figure 1.

The study was designed in accordance with the ethics guidelines of the 1975 Declaration of Helsinki and was approved by the Ethics Committee of Semmelweis University (#97/2012). Based on the study design and its retrospective nature, the Ethical Committee of Semmelweis University, Budapest, has waived the informed consent procedure.

### Detection of *H. pylori* Positivity in Biopsy Samples

Gastric biopsy samples were immediately immersed and fixed for 12–36 h in 10% buffered formalin before paraffin embedding. During the diagnostic work-up, H&E and Alcian Blue-Periodic Acid-Schiff stains were used for the histological diagnosis. Gastric structural alterations were assessed according to the updated Sydney system (22). Modified Giemsa staining and/or *Helicobacter* immunohistochemistry (carried out on Ventana Benchmark automated staining system / Ventana Medical Systems Inc., Tucson, AZ, United States / with B0471 polyclonal rabbit anti-*H. pylori* primary antibody / Dako, Glostrup, Denmark/) were used for the detection of *H. pylori* infection. The bacterial density (HP-density) was categorized semi-quantitatively in accordance with the updated Sydney system as follows: mild (1+, low density, scattered distribution), moderate (2+, medium density with small groups of bacteria) and severe (3+, high density with large groups of bacteria) (22). Each *H. pylori-*positive biopsy specimen was processed for further analysis of Cla-resistance.

### Fluorescence *in situ* Hybridization

*H. pylori* Cla-resistance was detected by BACTFish *H. pylori* Combi Kit, as previously described by Kocsmár et al. (18). Briefly, 4 μm thick sections were cut from formalin-fixed, paraffin-embedded gastric biopsy specimens. Subsequently, these were deparaffinized and heat pretreated by microwaving at 400 W for 20 min in Vector Antigen Unmasking Solution (Cat. No. H-3300, Vector Laboratories). The tissue sections were then hybridized for 90 min at 46 °C with DNA Probe Mix of BACTFish *H. pylori* Combi Kit. During this process, *H. pylori* bacteria were labeled with *H. pylori*-specific probe (Hpy-1, green fluorochrome FITC/fluorescein-isothiocyanate), which targets the 16S-rRNA of the bacterial cells. The probe cocktail also contained probes targeting the three most common Cla-resistance point mutations (ClaR1 [A2143G], ClaR2 [A2144G] and ClaR3 [A2143C], orange-red fluorescent Cy3) in the 23S-rRNA of the bacteria.

Specific fluorescent signals were examined under fluorescence microscope Leica DMRXA (Leica, Wetzlar, Germany) equipped with a longpass DAPI filter for blue nuclear staining, (Leica, Wetzlar, Germany) as well as with Spectrum Green and Spectrum Orange bandpass filters (Vysis, Downers Grove, IL, United States) for green and orange-red fluorescence, respectively. Images were taken using Leica DFC365 FX camera and documented by Leica CW4000 FISH analysis software.

**FIGURE 2:**
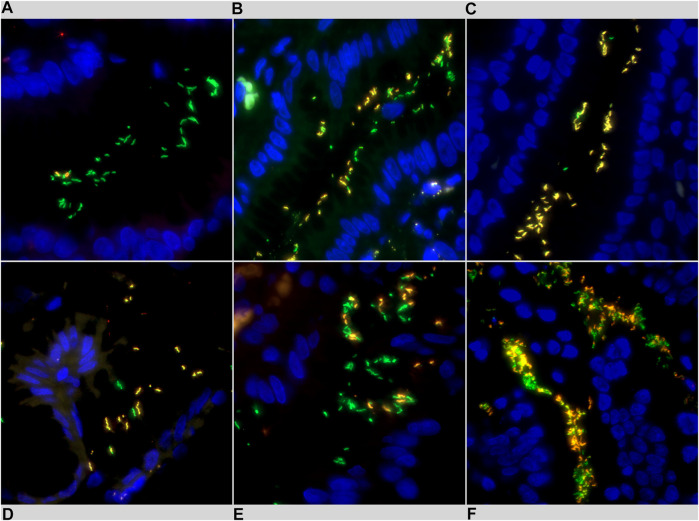
Representative images of *Helicobacter pylori* intraniche heteroresistance detected using fluorescence *in situ* hybridization (FISH). Clarithromycin susceptible *H. pylori* bacteria exhibit green fluorescence, clarithromycin resistant *H. pylori* bacteria appear in yellow. **(A)** low R-fraction, **(B)** moderate R-fraction, **(C)** high R-fraction, **(D)** low bacterial density (1+), **(E)** moderate bacterial density (2+), **(F)** high bacterial density (3+).

Prokaryotes like *H. pylori* are rich in ribosomes which exist in randomly distributed manner, thus by applying fluorescently labeled probes that target their rRNAs, the entire shape of the bacteria can be visualized by detecting their fluorescence with multi-channel imaging (23). Consequently, Cla-susceptible *H. pylori* appear as a green bacterium on fluorescent microscopy images. Meanwhile, Cla-resistant *H. pylori* bacteria display yellow fluorescence on multichannel composite images because of the mixed, green *H. pylori* specific probe signal and the red Cla-resistance specific probe signal (Figure 2).

### Process of Digital Morphometric Measurements

From each biopsy specimen, one or more areas with representative bacterial count and Cla-resistance status were chosen from fluorescent multichannel composite images obtained with ×63 objective, (HC PL Fluotar ×63/0,90 CORR, Leica, Wetzlar, Germany) and used as “representative areas” for morphometric purposes. The selection and annotation of the *H. pylori* bacteria were carried out using Photoshop CS3 (version 10, Adobe Systems, San José, CA, United States). All fluorescently labeled (including both susceptible/green and resistant/yellow) bacteria were identified and annotated into a selection channel. Subsequently, Cla-resistant bacteria were selected from the annotated images and saved into a separate selection channel. To quantify the area of the bacteria, the annotated selection of each image was copied into Image J (1.47v, National Institutes of Health, Bethesda, MD, United States), and fluorescence was measured individually in pixels using the pixel value statistics function on Image J. This method allowed us to quantify the bacterial area on the tissue slide by excluding all non-specific fluorescence. Furthermore, by annotating the whole bacterial population and the resistant bacteria separately, we were able to calculate the proportion of Cla-resistant bacteria in each image, thus obtaining the overall resistance fraction within the Cla-heteroresistant cases.

Finally, in order to assess the number of bacteria, five bacteria with fully visible longitudinal cross section were selected within the *H. pylori* population of each sample. Subsequently, we measured the area of the selected bacteria individually and averaged the results of these five measurements to get the typical size (area) of a single *H. pylori* in the given sample. In the next step, the total measured fluorescence area, the total green fluorescence area, and the total yellow fluorescence area were divided by the average size of a single *H. pylori* (specific for each sample), respectively, yielding the approximate number of susceptible and resistant *H. pylori* bacteria within each sample.

### Investigated Parameters

Proportion of the resistant bacteria in the *H. pylori* population was defined as “R-fraction”. Patients were also grouped according to the absolute number of counted *H. pylori* bacteria in all representative areas of a given case (HP-quant). The HP-quant is a three graded system (HP-quant 1/2/3) in which the three groups correspond to the three quartile of the cases (Q1—lower 25% [number of the counted bacteria is low], Q2—middle 50% [number of the counted bacteria is moderate], Q3—upper 25% [number of the counted bacteria is high]), according to the absolute number of the bacteria counted in the individual cases.

On the other hand, intrapatient variance was introduced to compare the variance in the R-fractions of representative areas among the individual patients. For this reason, R-fractions of the different images of a given patient (having more than one representative areas) were considered as an individual collection of data values and the variances of these “elementary data groups” (=patients) were calculated and compared within each subgroup of the patients. This method revealed the intrapatient variances among the representative areas.

With the aim to find one representative area of a given case we selected the area of the highest number of bacteria in each case and assigned it as ‘hot spot area’. Patients were categorized into subgroups according to the number of their measured representative areas (2 areas, 3 areas, and ≥4 areas). Random effects meta-regression analysis was performed to estimate the plausibility of the proportion of resistant and susceptible bacteria between the hot spot area and the total measured area of the same patient. The Risk Ratio method was used to evaluate the tendency of inaccuracy when using only the hot spot area for estimating the R-fraction of the given patient. Furthermore, the I^2^ statistics and Q-test were used to estimate the heterogeneity among the patients in different subgroups and the overall study.

### Statistical Analysis

During the statistical analysis, continuous variables were described as means, range and SD, and categorical variables as frequencies. All statistical analyses were carried out in R software environment (version 3.5.1.). Levine test was used to compare the variance of R-fraction between the matched subgroups and among the representative areas within a subgroup (Levine test with multiple groups). Forest plot for hot-spot analyses was performed using the ‘metafor’ package in R software environment (24). Cochrane Q-test and I^2^ statistics were used to measure heterogeneity within subgroups and among all subgroups. All statistical analyses were two-sided and *p* values were considered significant when *p* <0.05. All confidence intervals were calculated for 95% confidence.

## Results

### General Characteristics of the Cohort

Among the included patients, four cases (6.45%) were categorized into the A1 subgroup, which represents patients with one previous Cla-containing eradication therapy. Four patients (6.45%) were categorized into the A2 subgroup representing patients with two or more previous eradication attempts, while B1 and B2 represent the subgroups that have never had previous eradication. Among the latter subgroups, B1 subgroup with 32 patients (59.26%) showed successful eradication with Cla-containing regimen whereas B2 subgroup with 22 patients (40.74%) showed unsuccessful eradication with Cla-containing regimen, thus demonstrating the efficacy of Cla-based regimen in treating Cla-heteroresistant cases (Table 1).

**TABLE 1:** Cohort characteristics and histopathologic findings.

Characteristics of patient subgroups with clarithromycin-containing *H. pylori* eradication treatment		Total	A1 one prior unsuccessful eradication	A2 multiple prior unsuccessful eradications	B1 successful eradication after biopsy	B2 unsuccessful eradication after biopsy
Total	*n* (%)	62 (100%)	4 (6.45%)	4 (6.45%)	32 (59.26%)	22 (40.74%)
Age (year)	mean, SD	56 (SD: 16.86)	53.5 (SD: 16.78)	63.5 (SD: 20.21)	60 (SD: 16.41)	53 (SD: 17.68)
Sex	Male	21 (33.87%)	1 (25%)	1 (25%)	10 (31.25%)	9 (40.91%)
	Female	41 (66.13%)	3 (75%)	3 (75%)	22 (68.75%)	13 (59.09%)
HP-density	1+	12 (19.35%)	1 (25%)	0 (0%)	8 (25%)	3 (13.64%)
	2+	34 (54.84%)	2 (50%)	2 (50%)	20 (62.5%)	10 (45.45%)
	3+	16 (25.81%)	1 (25%)	2 (50%)	4 (12.5%)	9 (40.91%)
Histopathology	Active chronic gastritis	54 (87.1%)	4 (100%)	4 (100%)	26 (81.25%)	20 (90.91%)
	Intestinal metaplasia	9 (14.52%)	1 (25%)	0 (0%)	7 (21.88%)	1 (4.55%)
	Polyp	2 (3.23%)	0 (0%)	0 (0%)	1 (3.13%)	1 (4.55%)
	Peptic ulcer	4 (6.45%)	0 (0%)	0 (0%)	4 (12.5%)	0 (0%)
	Erosions	13 (20.97%)	1 (25%)	0 (0%)	5 (15.63%)	7 (31.82%)

The majority of the successfully eradicated cases (B1 subgroup) had a bacterial density of 1+ or 2+, 8 cases (25%) and 20 cases (62.5%) respectively, compared to the unsuccessfully eradicated cases (B2 subgroup), which had a bacterial density of 2+ or 3+, 10 cases (45.45%) and 9 cases (40.91%) respectively. Finally, the histopathological assessment suggested that 54 cases (87.1%) had active chronic gastritis. Other histopathological parameters investigated were intestinal metaplasia, polyps, peptic ulcers and erosions. Descriptive statistics of the included cases are shown in this table.

### R-Fraction Among Different Subgroups of Patients

**FIGURE 3:**
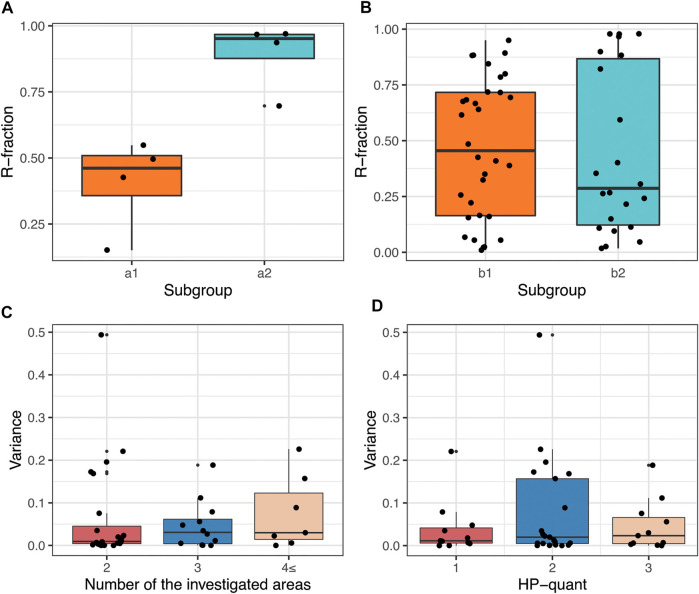
Distribution of resistant bacteria in Cla-heteroresistant *H. pylori* infections. **(A)** Boxplot representation of the mean R-fraction of the previously eradicated patients (a1: patients underwent one Cla-containing eradication attempt, a2: patients underwent multiple eradication attempts containing at least one Cla-based eradication attempt). **(B)** Boxplot representation of R-fraction in patients without any prior eradication attempt (b1: Successful eradication after diagnosis, b2: unsuccessful eradication attempt after diagnosis). **(C)** Boxplot showing that the greater the number of representative areas there are, the higher the variance of R-fraction there is (Examined in all the cases of the cohort having more than one representative area.). **(D)** Boxplot showing the distribution of the variance of R-fraction among representative areas in cases with different amount of counted *H. pylori* bacteria (HP-quant) (Examined in all the cases of the cohort having more than one representative area).

The average number of bacteria documented in the representative areas ranged from 138.3 to 168.7 among the different subgroups, with an average of 151.7 bacteria per patient (Table 2). The total average number of representative area was 2.27. There was a significant difference (*p* = 0.030, Mann Whitney–Wilcoxon test, Figure 3A) between the mean resistance fraction in subgroup A1 (40.54%) and A2 (89.24%), which may indicate that multiple eradication therapies can indirectly select the Cla-resistant clones. The difference of R-fraction between subgroups B1 (46.95%) and B2 (44.12%) was not significant, concluding that eradication failure is not in relation to the proportion of resistant bacteria in heteroresistant infections. (Table 2; Figure 3B). Linear regression also confirmed a significant difference when comparing subgroups A1 and A2 (*p* = 0.004), while subgroups B1 and B2 did not differ significantly (*p* = 0.761).

**TABLE 2:** Results of the quantitative measurements.

		Total	A1	A2	*p*	B1	B2	*p*
Total number of *H. pylori*	Mean, SD	151.7053 (SD: 171.4261)	160.895 (SD: 91.59)	156.42 (SD: 125.53)	–	138.28 (SD: 170.71)	168.70 (SD: 195.69)	–
Number of representative areas	Mean, range	2.27 (1–14)	2.5 (2–4)	2.75 (2–3)	–	2.16 (1–4)	2.32 (1–14)	–
R-fraction	Mean, SD	0.4826 (SD: 0.33)	0.4054 (SD: 0.18)	0.8924 (SD: 0.13)	**0.03038**	0.4695 (SD: 0.31)	0.4412 (SD: 0.37)	0.8672
Intergroup variances between resistant ratio	Variance, CI	0.1102 (0.0928–0.1394)	0.031 (0.001–5.196)	0.017 (0.0001–8.0424)	0.66	0.097 (0.076–0.141)	0.135 (0.098–0.2235)	0.6489
Intrapatient variances among representative areas	Variance mean, range	0.06 (4.34–07–4.94–01)	0.028 (0.0069–0.0755)	0.072 (0.00056–0.16857)	a1: 0.2352	0.07872 (6.47 × 10^−5^–4.94 × 10^−1^)	0.0311 (4.34 × 10^−7^–2.21 × 10^−1^)	**b1: 0.02517**
					a2: 0.0642			**b2: 0.01507**

H. pylori, Helicobacter pylori; CI, confidence interval; R-fraction, proportion of resistant bacteria in heteroresistant infection. Significant results are highlighted in bold.

### Intergroup Variances of R-Fraction and Intrapatient Variances of Representative Areas

Variance of R-fraction was calculated in ‘intergroup’ and in ‘intrapatient’ settings. Intergroup variance measured the variance of the patient’s mean R-fractions between the subgroups (A1 vs. A2 and B1 vs. B2). Neither of the subgroup analyses had significant difference (Table 2). Intrapatient variance was not significant in subgroups A1 and A2, suggesting that the distribution of resistant bacteria among the susceptible bacteria in patients after eradication therapy is more homogenous. In subgroups B1 and B2 however, the variance among the representative areas were significantly different (*p* = 0.025 and *p* = 0.015, respectively; Table 2). These results suggest the patchy distribution of resistant bacteria within the whole bacterial population in the case of previously non-eradicated heteroresistant cases.

Figure 3C demonstrates that intrapatient variance increases by the number of investigated areas. As well as, no relationship was observed between the numbers of the *H. pylori* bacteria counted in the case (HP-quant) and the intrapatient variance of the R-fraction (Figure 3D). These results suggest that the observed high intrapatient variances of the R-fractions is not due to sampling error, instead it is a special feature of the heteroresistant infections.

### R-Fraction of ‘Hotspot Areas’ is not Representative for the Total Measured Area

Patients having more than one representative area were investigated to find a single ‘hotspot area’ representative for the total measured area of the given case.

**FIGURE 4:**
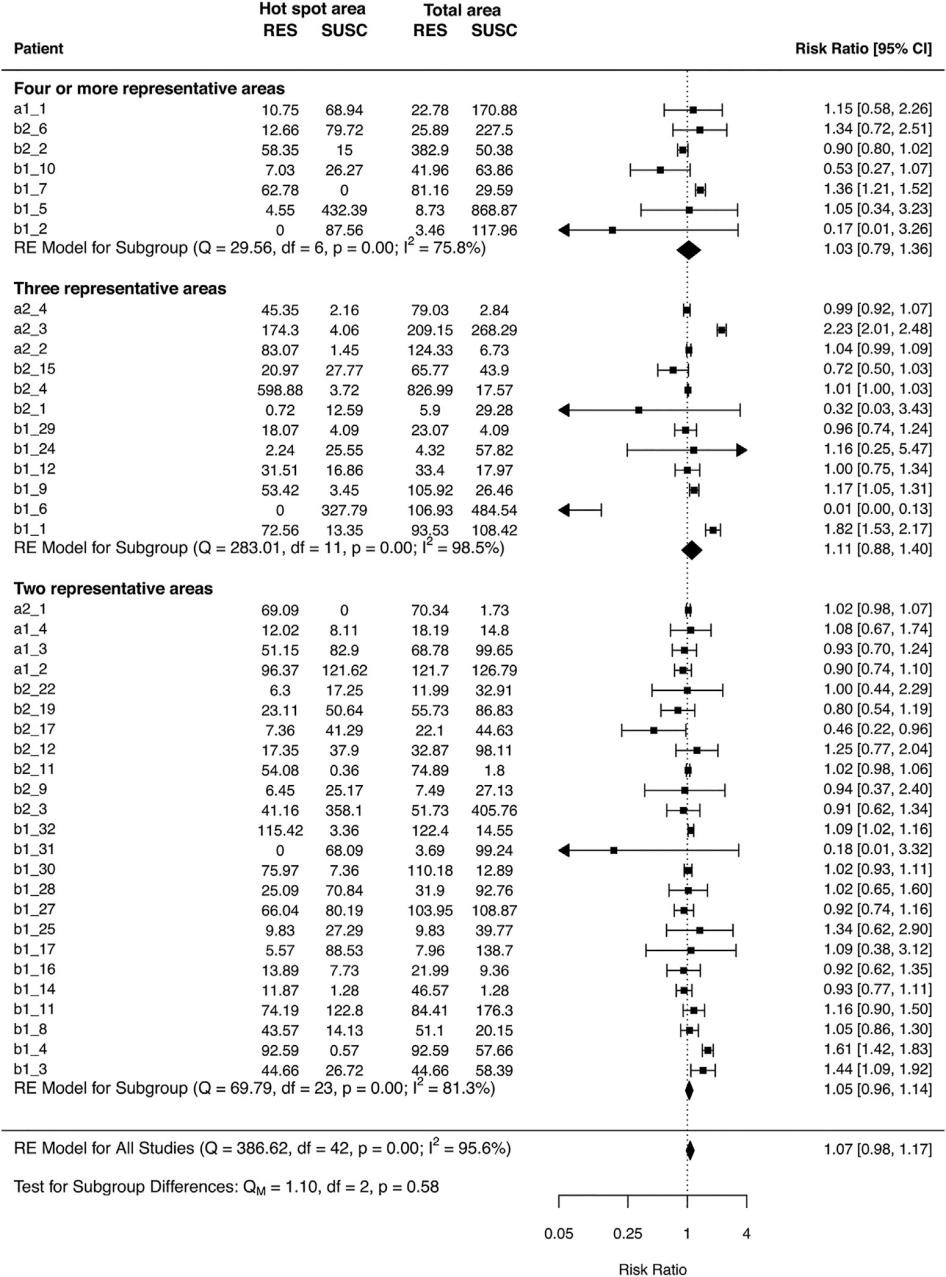
Hot spot analysis with different number of representative areas. Patients are labeled individually starting with the subgroup and the number of each case. Error bars depict 95% confidence intervals derived from random effects meta-analysis. Diamonds represents the pooled estimates with 95% confidence intervals based on random effects model for each group with different number of representative areas and for the three different group differences. RE = random effects model, RES = number of resistant bacteria, SUSC = number of susceptible bacteria.

According to the results of the I^2^ statistics, considerable heterogeneity was observed in patients within each subgroup (I^2^ > 75%) (25). High intragroup heterogeneity is also supported by the Q-test for each subgroup (*p* <0.0001). Subgroup differences were tested with Q-statistics, comparing the intragroup heterogeneity of the subgroups with each other. Test for subgroup differences was not significant, supporting the similarly high heterogeneity between the hot spot area and the total measured area, independently from the number of the representative areas. The results of the analysis are shown on Figure 4.

### HP-Density has a Predictive Role in Eradication Success

**FIGURE 5:**
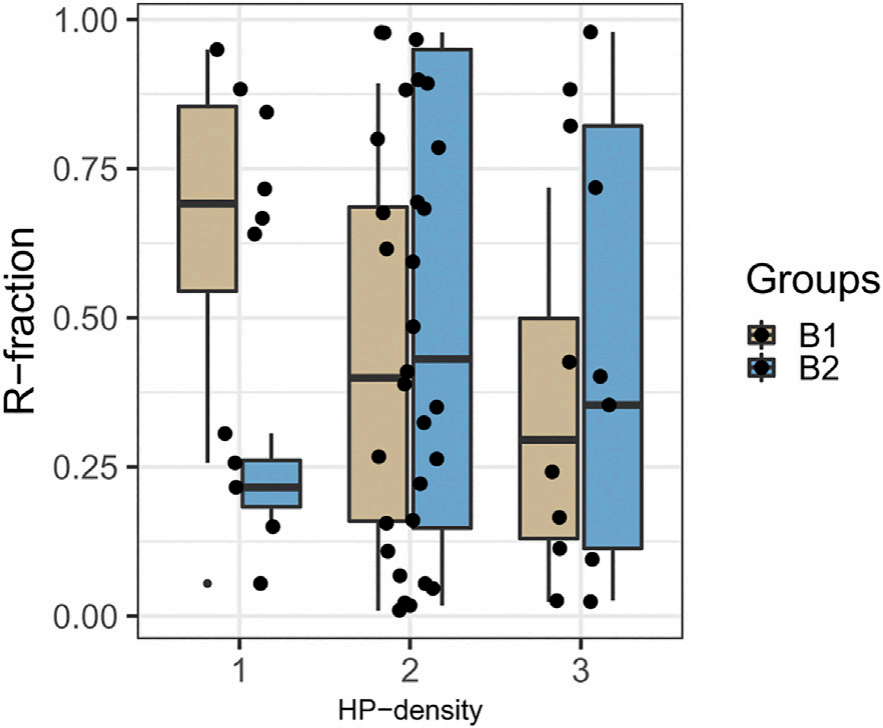
Proportion of resistant bacteria in cases with different HP-density. Boxplot represents the distribution of R-fractions according to the HP-density in cases with successful eradication (B1, light brown) and in cases with eradication failure (B2, blue). R-fraction = proportion of resistant bacteria in heteroresistant infections, HP-density = semi-quantitatively assessed bacterial density (1+/2+/3+).

On the jittered box plot shown in Figure 5, we visualized the therapy outcome (subgroups B1—successful eradication and B2—unsuccessful eradication) in relation to the R-fraction and the semi-quantitative HP-density (1+, 2+, 3+). Applying binary logistic regression analysis, we obtained significant negative correlation (*p* = 0.0392, OR 2.62, CI 1.05–6.55) between the therapy outcome and the HP-density, but not between the therapy outcome and R-fraction. This strongly suggests that the eradication success depends on the total volume of the bacteria instead of the proportion of resistant bacteria (R-fraction) in Cla-heteroresistant infections. Namely, cases with lower HP-density showed higher eradication rate.

## Discussion

Cla-heteroresistance is frequent, but the currently used routine diagnostic susceptibility test methods (E-test, PCR, or the combination) are set to diagnose interniche heteroresistance only, which may undermine the actual heteroresistance rates (17,18,20,26-30). In a previous study of our group, by applying FISH technique to gastric biopsy specimens, we demonstrated that the heteroresistance occurs in more than half (52%) of the Cla-resistant *H. pylori* infections (18). The most common form was found to be the intraniche heteroresistance occurring simultaneously in the antrum and the corpus of the stomach. In view of this, the interniche‐only detection approach could result in a reduction of heteroresistance rate by more than 50% (18). Despite the frequent occurrence of heteroresistant infections, no standard protocol for diagnosing intraniche heteroresistance, or treatment guidelines for *H. pylori* heteroresistant infections has been laid out yet (21).

In this retrospective study, we assessed the Cla-heteroresistant *H. pylori* infections using FISH technique for visualization, and digital morphometry for the measurement of resistance fractions. The resistant bacteria exhibited a patchy distribution among the susceptible ones. Interestingly, the efficacy of the Cla-containing eradication therapy depended on the total volume of the bacterial population rather than the R-fraction of the bacteria among the heteroresistant cases.

Cla-based triple therapy continues to be recommended as evidence-based first line therapy in North America and Europe for *H. pylori* infections (12,31). However, no optimal therapeutic strategy has been established for heteroresistant infections yet, therefore, Cla-resistance is managed uniformly in the everyday gastroenterology practice, independently from its homo- or heteroresistant type. According to the published data in De Francesco et al. (32) and Kocsmár et al. (18), the heteroresistant infections represent a distinct, separate entity, with intermediate eradication success rate compared to the homoresistant and the susceptible infections. No such predictive factor however, has been identified so far, which can support the therapeutic decisions in the Cla-heteroresistant subgroup of *H. pylori* infections. We hypothesized that eradication success of a Cla-containing regimen depends on the proportion of susceptible and resistant bacteria in the *H. pylori* population. Namely, it seems rational that Cla-heteroresistant cases with lower R-fraction (lower proportion of Cla-resistant bacteria) would respond better to a Cla-based therapy. However, no significant difference was established when comparing Cla-resistant and -susceptible fractions between the subgroup B1 (successful *H. pylori* eradication) and B2 (unsuccessful eradication), indicating that the outcome of a Cla-based eradication regimen is not affected by the proportion of the Cla-resistant bacteria in patients with no previous eradication therapy. On the contrary, we saw a biological difference between subgroup A1 (previously one eradication attempt) and A2 (multiple eradication attempts). Namely, there was a significant increase in the overall proportion of resistance among the cases with multiple eradication history (89.2%) compared to the cases with only one eradication history previously, (40.5%) concluding that multiple eradication history can more effectively select the Cla-resistant clones.

Up to now, the Cla-based classic triple therapy is still the safest therapeutic option in *H. pylori* eradication, especially from the aspect of long-term effect on gut microbiota (33). This underlines the importance of selecting patients who would benefit from a Cla-containing eradication treatment. Our results support that heteroresistance is a distinct entity in which the success of eradication does not depend on the average resistance fraction, but that Cla-containing regimens were more effective in patients with lower bacterial load. Accordingly, these results can contribute to broaden the circle of those for whom a Cla-based regimen might be recommended.

This study provides further insights into the intragastric distribution of the *H. pylori*. In line with our previous experiences (18,34), both the *H. pylori* bacteria and their Cla-resistant subpopulation are distributed in a patchy manner on the gastric mucosa. Consequently, sampling error due to this random spatial distribution is unavoidable. Moreover, our hot spot analysis strongly suggests that the diversity between the different gastric areas is so high, which could not be simply evaded by increasing the number of the sampling sites. Of course, the higher the number of the investigated areas there are, the higher the chance of finding resistant bacteria in a Cla-heteroresistant *H. pylori* population. However, the sampling number of the mapping biopsy is limited during a gastroscopy. Our hot spot analysis indicated that the number of the routinely obtained biopsies is not enough to decrease the sampling error noticeably if only one field of view is investigated by microscopy. This is why it is so important to scrutinize the whole mucosal area of each tissue sample of a gastric biopsy case during the microscopic evaluation, both for diagnosing the *H. pylori* infection and to reveal the presence of Cla-resistant bacteria during the evaluation of the *H. pylori* Cla-resistance FISH.

The biological aspect of this random and patchy distribution of the resistant *H. pylori* bacteria is not sufficiently known. Bacteria of divergent resistance status within an *H. pylori* population can be originated from either the coexistence of susceptible and resistant isolates of the same *H. pylori* strain, or from the superinfection of an *H. pylori* strain by another one having distinct resistance status (18). Our previous results have shown that intraniche Cla-heteroresistance (simultaneous presence of susceptible and resistant *H. pylori* bacteria in the same gastric site) is much more frequent than the interniche heteroresistance (*H. pylori* subpopulations of different resistance status at different sites of the stomach) (18). Since the coexistence of distinct *H. pylori* strains is more closely associated with the interniche heteroresistance, it is much less common, especially in human populations with low *H. pylori* prevalence, where superinfection events are rare (35). Mixing of the susceptible and resistant *H. pylori* bacteria facilitates the horizontal gene transfer between them. In light of this, it seems that the spatial separation of the different *H. pylori* strains in the gastric environment may contribute to sustain their divergent genetic background, and therefore, their distinct entity. Naturally, this is only true in the absence of selection pressure from macrolide antibiotics like clarithromycin. Macrolide treatment probably increases the interstrain genetic interactions as well. As a Cla-based eradication attempt provides a very strong selection pressure to the *H. pylori*, Cla-susceptible bacteria will be selected out from a heteroresistant bacterial population, or Cla-resistance may emerge from spontaneous mutation in a susceptible strain. Multiple eradication attempts can further increase the R-fraction as we found a significantly higher mean value in subgroup A2 (cases with multiple eradication history) than is subgroup A1 (cases with only one previous eradication attempt).

As clarithromycin-containing eradication treatment was successful in about 60% of the Cla-heteroresistant cases, another question emerges: how can Cla act against the resistant bacteria of a Cla-heteroresistant *H. pylori* population? In this regard, it is important to note that Cla-based eradication regimens contain at least one other antibiotic and/or antibacterial chemotherapeutic agent, which can be effective against the Cla-resistant bacteria. Thus, not the Cla but this other component can be responsible for the anti-*H. pylori* activity of a Cla-containing regimen.

However, the reason why combination therapies are used for eradication purposes rather than monotherapy is because monotherapeutic approaches are less effective (36). This explains why the Cla-containing eradication combinations are more effective in Cla-heteroresistant cases of lower *H. pylori* density. Since only the non-claritromycin component is effective on Cla-resistant bacteria, efficacy of the combination is limited, especially when the bacterial load is high. Of course, other factors like pH increasing effect of the PPI component of the eradication regimen (37), or individual variations in the composition and thickness of the mucus layers of the stomach might also affect the eradication success rate. This underlines the need for identifying further predictive factors for the Cla-heteroresistant *H. pylori* infections.

Our finding suggesting that the eradication success of a Cla-containing regimen depends more likely on the *H. pylori* density/load of the Cla-heteroresistant *H. pylori* rather than the proportion of resistant bacteria (R-fraction) leads to another question: is clarithromycin treatment recommended in heteroresistant cases with low *H. pylori* load? It is important to emphasize that the eradication success rate was 73% in the 1+, 67% in the 2+, and only 31% in the 3+ *H. pylori* density cases. Accordingly, it is clear that Cla-based eradication is not recommended in cases with high bacterial load. However, the success rates of the 1+ and 2+ cases are relatively high, even if it is still lower than the desirable ≥90%. Therefore, Cla-based therapy is conceivable in such cases, but identification of further predictive factors would be necessary. Moreover, the accurate determination of *H. pylori* density in routine histological reports of gastric biopsies would also be essential.

One of the limitations of our study was its retrospective nature. Additionally, due to the low event number in the final subgroups after sequential grouping, certain comparisons lacked statistical power. Furthermore, the assessment method of the bacterial number considered the fully visible longitudinal cross section area of an average *H. pylori* as one bacterium. As the orientation of the bacteria is random in the sample, the number of the diagonally or perpendicularly oriented bacteria to the sectional plane was underestimated. However, since this phenomenon affects the individual images equally, it did not cause a bias in the intrapatient and intragroup comparisons. Moreover, as the FISH technique used only detects the three most frequent point mutations of 23S rRNA, the resistance caused by less common genetic mechanisms may have been missed (38–42). In addition, we did not have the information regarding the resistance to other antibacterial components (e.g. metronidazole) of the Cla-based eradication therapies of the patients in our cohort. Given the limited number of cases and the retrospective nature of the study, our results should be considered somewhat preliminary and will require further confirmation. Based on our previous publications, it is clear that heteroresistant *H. pylori* infections account for about half of all clarithromycin-resistant cases (18,38). Therefore, further prospective studies with increased patient case number would be necessary to establish a more definite relationship between eradication success and bacterial load, as well as, to find further predictive factors to facilitate therapeutic decisions, and in result contribute to establishing a standard protocol for Cla-heteroresistant infections.

To our knowledge, our study is the first which investigated the predictive role of the proportion of resistant bacteria in Cla-heteroresistant *H. pylori* infections, and successfully identified a predictive factor for Cla-based therapies. In conclusion, due to the patchy distribution of resistant bacteria among the susceptible population, multiple sampling is recommended to prevent false susceptible results. Also, our study demonstrated no significant correlation between the eradication success and the overall resistance fraction. Eradication of Cla-heteroresistant infections by Cla-containing regimens is more effective in patients with low *H. pylori* load, and thus bacterial density may be a useful predictive factor in aiding the therapeutic decisions of Cla-heteroresistant cases, as *H. pylori* density is routinely assessed in histological practice (22). The efficacy of Cla-based regimens in the treatment of Cla-heteroresistant infections is around 60%, highlighting the importance of investigating additional predictive factors for Cla-based eradication therapy in heteroresistant cases.

## Data Availability

The original contributions presented in the study are included in the article/Supplementary Material, further inquiries can be directed to the corresponding author.
