# Supplementary material for: Efficacy of Clarithromycin Depends on the Bacterial Density in Clarithromycin-Heteroresistant Helicobacter pylori Infections: An In Situ Detected Susceptibility and Quantitative Morphometry-Based Retrospective Study
Source: Pathol Oncol Res. 2021 Jun 29;27:1609863. doi: 10.3389/pore.2021.1609863 (PMC8275651; doi:10.3389/pore.2021.1609863)
Supplement: Supplementary file 1 [file DataSheet1.PDF]

Supplementary Material

Efficacy of clarithromycin depends on the bacterial density in clarithromycin-heteroresistant *Helicobacter pylori* infections - an in situ detected susceptibility and quantitative morphometry-based retrospective study

Jewel Ju Ea Kim, Ildikó Kocsmár, György Miklós Buzás, Ildikó Szirtes, Orsolya Rusz, Csaba Diczházi, Attila Szijártó, István Hritz, Zsuzsa Schaff, András Kiss, Éva Kocsmár and Gábor Lotz

Eradication history of patients from subgroups A1 (patients with one previous clarithromycin-based eradication attempt) and A2 (patients with multiple previous eradication attempts including at least one clarithromycin-based regimen) ***before*** the sampling date of the gastric biopsy investigated in the frame of the study.

**Patient A1/1**	**Drug**	**Dosage**		
**Eradication #1**	Rabeprazole	20 mg	b.i.d.	10 days
	Amoxicillin	1000 mg	b.i.d.	
	Clarithromycin	500 mg	b.i.d.	

**Patient A1/2**	**Drug**	**Dosage**		
**Eradication #1**	Rabeprazole	20 mg	b.i.d.	10 days
	Amoxicillin	1000 mg	b.i.d.	
	Clarithromycin	500 mg	b.i.d.	

**Patient A1/3**	**Drug**	**Dosage**		
**Eradication #1**	Rabeprazole	20 mg	b.i.d.	10 days
	Amoxicillin	1000 mg	b.i.d.	
	Clarithromycin	500 mg	b.i.d.	

**Patient A1/4**	**Drug**	**Dosage**		
**Eradication #1**	Rabeprazole	20 mg	b.i.d.	10 days
	Amoxicillin	1000 mg	b.i.d.	
	Clarithromycin	500 mg	b.i.d.	

**Patient A2/1**	**Drug**	**Dosage**		
**Eradication #1**	Rabeprazole	20 mg	b.i.d.	10 days
	Amoxicillin	1000 mg	b.i.d.	
	Clarithromycin	500 mg	b.i.d.	
**Eradication #2**	Rabeprazole	20 mg	b.i.d.	10 days
	Tetracycline	500 mg	t.i.d.	
	Tinidazole	500 mg	b.i.d.	
**Eradication #3**	Rabeprazole	20 mg	b.i.d.	10 days
	Amoxicillin	1000 mg	b.i.d.	
	Rabeprazole	20 mg	b.i.d.	10 days
	Tetracycline	500 mg	t.i.d.	
	Levofloxacin	500 mg	b.i.d.	

Patient A2/2	Drug	Dosage		
**Eradication #1**	Rabeprazole	20 mg	b.i.d.	10 days
	Amoxicillin	1000 mg	b.i.d.	
	Clarithromycin	500 mg	b.i.d.	
**Eradication #2**	Ranitidine bismuth citrate	400 mg	b.i.d.	14 days
	Tetracycline	500 mg	t.i.d.	8 days
	Tinidazole	500 mg	b.i.d.	8 days
**Eradication #3**	Pantoprazole	40 mg	b.i.d.	10 days
	Ciprofloxacin	500 mg	b.i.d.	
	Amoxicillin	1000 mg	b.i.d.	
**Eradication #4**	Rabeprazole	20 mg	b.i.d.	10 days
	Levofloxacin	500 mg	b.i.d.	
	Amoxicillin	1000 mg	b.i.d.	

Patient A2/3	Drug	Dosage		
**Eradication #1**	Rabeprazole	20 mg	b.i.d.	10 days
	Amoxicillin	1000 mg	b.i.d.	
	Clarithromycin	500 mg	b.i.d.	
**Eradication #2**	Ranitidine bismuth citrate	400 mg	b.i.d.	14 days
	Tetracycline	500 mg	t.i.d.	8 days
	Tinidazole	500 mg	b.i.d.	8 days

Patient A2/4	Drug	Dosage		
**Eradication #1**	Rabeprazole	20 mg	b.i.d.	10 days
	Amoxicillin	1000 mg	b.i.d.	
	Clarithromycin	500 mg	b.i.d.	
**Eradication #2**	Pantoprazole	40 mg	b.i.d.	10 days
	Tetracycline	500 mg	b.i.d.	
	Tinidazole	500 mg	b.i.d.	
